# Prognostic value of high-sensitivity cardiac troponin I early after coronary artery bypass graft surgery

**DOI:** 10.1186/s13019-022-02027-x

**Published:** 2022-11-01

**Authors:** Samuele Nanni, Mattia Garofalo, Matteo Schinzari, Elena Nardi, Franco Semprini, Paola Battistini, Francesco Barberini, Alberto Foà, Massimo Baiocchi, Andrea Castelli, Gianluca Folesani, Davide Pacini, Nazzareno Galiè, Anna Corsini

**Affiliations:** 1 Cardiology Unit, IRCCS Azienda Ospedaliero-Universitaria di Bologna, Bologna, Italy; 2 Department of Specialist, Diagnostic, and Experimental Medicine, Alma Mater Studiorum University of Bologna, Bologna, Italy; 3 Cardiothoracic and Vascular Anesthesia and Intensive Care Unit, IRCCS Azienda Ospedaliero-Universitaria di Bologna, Bologna, Italy; 4 Division of Cardiac Surgery, IRCCS Azienda Ospedaliero-Universitaria di Bologna, Bologna, Italy

**Keywords:** High-sensitivity cardiac troponin I, Coronary artery bypass graft, Periprocedural myocardial infarction, Left ventricular ejection fraction

## Abstract

**Background:**

The diagnosis of periprocedural myocardial infarction (PMI) after coronary artery bypass graft (CABG) is based on biochemical markers along with clinical and instrumental findings. However, there is not a clear cutoff value of high-sensitivity cardiac troponin (hs-cTn) to identify PMI. We hypothesized that isolated hs-cTn concentrations in the first 24 h following CABG could predict cardiac adverse events (in-hospital death and PMI) and/or left ventricular ejection fraction (LVEF) decrease.

**Methods:**

We retrospectively enrolled all consecutive adult patients undergoing CABG, alone or in association with other cardiac surgery procedures, over 1 year. Hs-cTn I concentrations (Access, Beckman Coulter) were serially measured in the post-operative period and analyzed according to post-operative outcomes.

**Results:**

300 patients were enrolled; 71.3% underwent CABG alone, 33.7% for acute coronary syndrome. Most patients showed hs-cTn I values superior to the limit required by the latest guidelines for the diagnosis of PMI. Five patients (1.7%) died, 8% developed a PMI, 10.6% showed a LVEF decrease ≥ 10%. Hs-cTn I concentrations did not significantly differ with respect to death and/or PMI whereas they were associated with LVEF decrease ≥ 10% (p value < 0.005 at any time interval), in particular hs-cTn I values at 9–12 h post-operatively. A hs-cTn I cutoff of 5556 ng/L, a value 281 (for males) and 479 (for females) times higher than the URL, at 9–12 h post-operatively was identified, representing the best balance between sensitivity (55%) and specificity (79%) in predicting LVEF decrease ≥ 10%.

**Conclusions:**

Hs-cTn I at 9–12 h post-CABG may be useful to early identify patients at risk for LVEF decrease and to guide early investigation and management of possible post-operative complications.

**Supplementary Information:**

The online version contains supplementary material available at 10.1186/s13019-022-02027-x.

## Introduction

Nowadays, ischemic heart disease remains the leading cause of morbidity and mortality worldwide [1]. Coronary artery bypass grafting (CABG) is an established option for coronary revascularization, and it is recommended in case of multivessel coronary artery disease or left main disease, especially when combined with high anatomical complexity or diabetes [2].

As any other type of cardiac surgery, CABG intervention can potentially cause periprocedural myocardial infarction (PMI) or myocardial injury, the latter one being defined as an isolated elevation of cardiac biomarkers without clinical and/or instrumental signs of an ischemic etiology [3]. In fact, the diagnosis of CABG-related (type 5) myocardial infarction (MI) does not rely only on the elevation of cardiac laboratory markers, but it also requires additional criteria such as new pathological Q waves on ECG, new graft or native coronary artery occlusion at angiography, or the imaging evidence of new loss of viable myocardium or of new regional wall motion abnormality in a pattern consistent with an ischemic etiology [4, 5].

Studies aiming at investigating the clinical significance and prognostic impact of PMI showed that its incidence and prognosis depend on the diagnostic criteria employed; nevertheless, PMI is generally associated with an increased risk of major cardiovascular events, after both percutaneous coronary intervention (PCI) and CABG, regardless of the definition applied [6–8]. After decades of cardiovascular research and clinical practice, the diagnosis of PMI after PCI and CABG, as suggested by both the Third and the Fourth Universal Definition of Myocardial Infarction, is still based on an arbitrary value of biochemical markers and there is not a clear cut off value to distinguish PMI from periprocedural myocardial injury [4, 5].

Cardiac troponin (cTn) is recommended as the preferred biomarker for the diagnosis of type 5 MI, due to its superior sensitivity and specificity compared to traditional biomarkers such as creatine kinase [3, 9]. Based on the magnitude of post-operative cTn I or cTn T elevation there exists a documented gradual increase in short and long-term mortality following CABG surgery, with a clear association between short and long-term mortality and isolated elevations of cTn T > 7 times of the upper reference limit (URL) and cTn I levels > 20 times of the URL [3]. High-sensitivity cardiac troponin (hs-cTn) assays have recently been developed, being more sensitive than previous assays at detecting lower troponin concentrations [9]; however, there exist several different assays and only few data are available about their application and augmented value in the diagnosis of type 5 MI [3].

Indeed, it is a widespread routine, also in use in our Institution, to measure serial hs-cTn concentrations in the post-operative period to assess the degree of myocardial injury and, depending on the clinical scenario, eventually make a diagnosis of PMI.

The aim of our study was to explore the clinical significance and the prognostic value of the serial testing of hs-cTn I in the first 24 h after CABG intervention, alone or in association with other cardiac surgery procedures.

In particular, we hypothesized that isolated hs-cTn I concentrations in the first 24 h following CABG surgery could predict main cardiac adverse events (in-hospital death and PMI) and/or left ventricular ejection fraction (LVEF) modifications (LVEF decrease ≥ 10%).

## Methods

### Ethics approval and consent to participate

The study was approved by the local Ethics Committee (Comitato Etico Area Vasta Emilia Centro).

All methods were performed in accordance with the Declaration of Helsinki as well as with our institutional ethics principles, guidelines and regulations.

Given the observational and retrospective nature of the study, patients’ consent was inferred in line with the general consent expressed upon hospital admission and, as requested by the ethics committee, specifically updated at the first medical re-contact with the patient, if any.

### Study population, data collection and definitions

We retrospectively enrolled all consecutive patients ≥ 18 years old who underwent CABG, alone or in association with other cardiac surgery/surgery on the ascending aorta, at S. Orsola-Malpighi Hospital from September 1, 2018, to September 1, 2019.

We analyzed each patient’s clinical record and collected demographic data as well as recent and past medical history, including the presence of cardiovascular risk factors and cardiovascular events such as previous MI, previous percutaneous and/or surgical coronary revascularization, previous cardiac surgery, and previous episodes of heart failure. Moreover, we gathered data about patients’ comorbidities, including the presence of peripheral artery disease, symptomatic chronic lung disease, chronic renal disease, and history of cerebrovascular disease (previous stroke and/or transient ischemic attack). Most of such variables were defined according to the European System for Cardiac Operative Risk Evaluation (EuroScore) II risk model, which we also calculated for each patient [10]. In particular: acute coronary syndromes were defined according to the related current guidelines [11]; previous MI was categorized as recent if occurring within the past 90 days before the date of current surgery; dyspnea and angina were graded according respectively to the New York Heart Association and to the Canadian Cardiovascular Society classification systems [12, 13]; previous heart failure was defined as a previous hospitalization for heart failure and/or signs/symptoms of heart failure and/or left ventricular ejection fraction ≤ 35% [14]; peripheral artery disease was defined as extra-cardiac arteriopathy manifested as any among lower limb claudication, carotid occlusion or > 50% stenosis, amputation due to arterial disease, previous or planned intervention on the abdominal aorta, limb arteries or carotids; symptomatic chronic lung disease was defined as long term use of bronchodilators or steroids for lung disease; poor mobility was defined as severe impairment of mobility secondary to musculoskeletal or neurological dysfunction with need of walking aid; the estimated glomerular filtration rate (eGFR) was calculated via the Cockcroft-Gault formula [15]; endocarditis was considered active when patients were still on antibiotic treatment for endocarditis at the time of surgery.

We also collected data on the surgical intervention. Surgical coronary revascularization was performed as indicated by current guidelines [2]. Based on the degree of urgency, surgery was classified as follows: salvage surgery in the case of need for cardiopulmonary resuscitation en-route to the operating theatre or before induction of anesthesia, emergency surgery in the case of need for operation before the beginning of the next working day after decision to intervene has been taken, urgent surgery in the case of all other patients whose admission and surgery were not scheduled, and elective surgery in the case of routine admission for operation. Critical preoperative state was defined as the pre-operative presence of any among ventricular tachycardia, ventricular fibrillation, aborted sudden death, cardiopulmonary resuscitation, ventilation (before entry into the anesthetic room), hemodynamic support by means of inotropes, intra-aortic balloon pump (IABP) or ventricular assist device, or acute renal failure (new-onset anuria or oliguria).

Furthermore, pre-operative and post-operative laboratory findings were reviewed, including hs-cTn I, along with pre-operative and post-operative electrocardiographic and echocardiographic findings, as well as post-operative angiographic findings when available.

Finally, post-operative events, including death, stroke (ischemic or hemorrhagic), reintervention, need of circulatory support (IABP and/or extracorporeal membrane oxygenation, ECMO), renal replacement therapy, final diagnosis of PMI, and total hospitalization length were recorded.

In line with the Fourth Universal Definition of Myocardial Infarction [5] CABG-related PMI was defined as an elevation of hs-cTn I values > 10 times the 99^th^ percentile Upper Reference Limit (URL) in patients with normal baseline hs-cTn I values; for patients with known elevated but stable (≤ 20% variation) or falling baseline hs-cTn I, post-procedural hs-cTn I must be > 10 times the 99^th^ percentile URL and rise by > 20% with respect to the pre-procedural value. An additional criterion was also required among the development of new pathological Q waves on electrocardiogram (ECG), the imaging evidence of new loss of viable myocardium or of new regional wall motion abnormality in a pattern consistent with an ischemic etiology, or the finding of new graft or native coronary artery occlusion at angiography.

### High-sensitivity cardiac troponin I measurements

Cardiac troponin values had been repeatedly measured at fixed time intervals after surgery by using the Access hs-cTn I assay by Beckman Coulter. The 99^th^ percentile URL established in a population of healthy adults were, respectively, 11.6 ng/L for females and 19.8 ng/L for males [16].

### Clinical outcomes

The prespecified clinical outcomes of the study were represented by in-hospital death or PMI, in-hospital death, PMI, and LVEF decrease ≥ 10% after CABG surgery. In particular, we investigated the power of hs-cTn I in predicting the above-mentioned clinical outcomes.

### Statistical analyses

Continuous variables are presented as means ± standard deviations (SD) or medians and interquartile ranges (IR), as indicated depending on the normality of the distribution, while categorical variables are presented as numbers and percentages. The non parametric Wilcoxon rank-sum test was applied in order to compare continuous variables while the Chi-squared test was applied in order to compare categorical variables.

Univariable logistic regression analysis was used to evaluate the unadjusted association between LVEF decrease ≥ 10% after CABG surgery as this was the only outcome which resulted significantly associated with hs-cTn I values in our study population. The following variables were examined at univariable logistic analysis as significantly associated with LVEF decrease ≥ 10%: hs-cTn I in the first 24 h following CABG, previous cardiac surgery, number of surgical procedures, cardiopulmonary bypass time, aortic cross clamp time, new regional wall motion abnormalities at post-operative echocardiogram, and PMI.

Receiver-operating characteristic (ROC) curves of the post-operative hs-cTn I values identified as predictor of LVEF decrease ≥ 10% were performed to evaluate the diagnostic performance in terms of discrimination power [area under the receiver operator characteristic curve (AUC)]. The Youden index was then used to identify the optimal cut-off value.

P-values less than 0.05 were deemed statistically significant.  

All analyses were performed with IBM SPSS Statistics package for Windows, version 25.0 (BM Co., Armonk, NY, USA).

## Results

### Study population

A total of 300 patients undergoing CABG alone or in association with other cardiac surgery at the tertiary hospital S. Orsola-Malpighi (Bologna, Italy) from September 1, 2018, to September 1, 2019, were enrolled and represented the study population.

Baseline clinical characteristics of the study population are showed in Table 1.

**Table 1:** Baseline clinical characteristics of the study population

	Overall N = 300
*Demographics and past medical history*	
Age, years, median [Q1-Q3]	70 [63–76]
Males, n (%)	253 (84.3)
BMI, kg/m^2^, median [Q1-Q3]	27 [24–30]
Hypertension, n (%)	236 (78.7)
Hypercholesterolemia, n (%)	233 (77.6)
Diabetes, n (%) On insulin, n (%)	95 (31.7) 14 (4.7)
Smoker	
Current, n (%)	45 (15)
Previous, n (%)	137 (45.7)
Previous myocardial infarction, n (%)	58 (19.3)
Previous PCI/CABG, n (%)	47 (15.7)
Previous episode of congestive heart failure, n (%)	43 (14.3)
Previous stroke/TIA, n (%)	23 (7.7)
Peripheral arterial disease, n (%)	58 (19.3)
Symptomatic chronic lung disease, n (%)	18 (6)
Dialysis, n (%)	4 (1.3)
*Pre-operative clinical characteristics*	
Hospitalization for ACS, n (%)	101 (33.7)
Left main stenosis > 50%, n (%)	73 (24.3)
Three-vessel disease, n (%)	147 (49)
LVEF, %, median [Q1-Q3] ≥ 50%, n (%) 40–49%, n (%) < 40%, n (%)	60 [51–65] 243 (81) 40 (13.3) 17 (5.7)
Creatinine, mg/dl, median [Q1-Q3]	0.96 [0.8–1.12]
Estimated GFR by Cockcroft-Gault, ml/min, mean ± SD	82 ± 32
*Other pre-operative risk assessment parameters*	
Reduced mobility, n (%)	0 (0)
Critical pre-operative status, n (%)	4 (1.3)
NYHA class dyspnea	
I, n (%) II, n (%) III, n (%) IV, n (%)	174 (58.0) 81 (27.0) 32 (10.7) 13 (4.3)
CCS Class IV angina, n (%)	55 (18.3)
Previous cardiac surgery, n (%)	3 (1)
Active IE, n (%)	3 (1)
MI in the previous 90 days, n (%)	63 (21)
Estimated PASP	
≤ 30 mmHg, n (%) 31–55 mmHg, n (%) > 55 mmHg, n (%)	268 (89.3) 29 (9.7) 3 (1)
Non-elective operation, n (%)	131 (43.7)
EuroScore II estimated risk of in-hospital mortality, mean ± SD	2.9 ± 4.8
*Main indication for surgical treatment*	
CAD, n (%) AS, n (%) AR, n (%) MR, n (%) CAD + AR, n (%) CAD + MR, n (%) IE, n (%) Other^#^, n (%)	223 (74.3) 43 (14.3) 15 (5) 10 (3.3) 1 (0.3) 2 (0.7) 3 (1) 3 (1)

^#^ 1 patient with left ventricular pseudoaneurysm; 1 patient with suspected free wall rupture with cardiac tamponade; 1 patient with concomitant aortic aneurysm + severe aortic regurgitation + triple vessel coronary artery disease

ACS: acute coronary syndrome; AR: aortic regurgitation; AS: aortic stenosis; BMI: body mass index; CABG: coronary artery bypass grafting; CAD: coronary artery disease; CCS: Canadian Cardiovascular Society; GFR: glomerular filtration rate; IE: infective endocarditis; LVEF: left ventricular ejection fraction; MI: myocardial infarction; MR: mitral regurgitation; NYHA: New York Heart Association; PASP: pulmonary artery systolic pressure; PCI: percutaneous coronary intervention; SD: standard deviation; TIA: transient ischemic attack

The median age was 70 (IR 63–76), the majority of patients were males (253 patients, 84.3%). Arterial hypertension was present in 236 patients (78.7%), about one third were diabetic (95 patients, 31.7%), the mean eGFR was 82 ± 32 ml/min, and only 4 (1.3%) were on dialysis. Fifty-eight patients (19.3%) had a history of MI, 47 (15.7%) had previously received percutaneous (44, 14.7%) or surgical (3, 1.0%) myocardial revascularization. Median pre-operative LVEF was 60% (IR 51–65). CABG treatment was performed during hospitalization for an acute coronary syndrome (ACS) in 101 cases (33.7%), it was carried out as a surgical urgency in 131 (43.7%) and with critical pre-operative conditions in 4 (1.3%). The mean EuroScore II value was 2.9 ± 4.8.

Coronary artery disease represented the main indication for surgery in most cases (223, 74.3%); in particular, left main stenosis > 50% was present in 73 patients (24.3%), and three-vessel disease in 147 (49%). Heart valve disease was the main indication for most of the other patients (68, 22.7%), while in few remaining cases (9, 3.0%) surgery was performed for either mixed or different surgical indication, including infective endocarditis, as detailed in Table 1.

### Cardiac surgery

Peri-operative details are listed in Table 2. Two hundred fourteen interventions (71.3%) consisted in CABG only; 74 (24.7%) in CABG and valve repair/replacement (mainly aortic valve surgery) without involvement of the ascending aorta; 11 (3.7%) in CABG, valve and concomitant surgery on the ascending aorta; 1 (0.3%) in CABG and ventricular aneurysmectomy. Isolated CABG was mainly performed on-pump (207 out of 214 cases, 96.7%), with a median cardio-pulmonary bypass time of 106 min (IR 78–133). Up to 6 anastomoses were performed, but most commonly 1 (67, 22.3%), 2 (125, 41.7%), 3 (74, 24.7%) or 4 anastomoses (28, 9.3%).

**Table 2:** Type of intervention, operative and post-operative details

	Overall N = 300
*Type of cardiac intervention*	
CABG, n (%)	214 (71.3)
CABG + AVR, n (%)	57 (19.0)
CABG + AVR + MVR, n (%)	4 (1.3)
CABG + AVR + tricuspid valve repair, n (%)	1 (0.3)
CABG + MVR, n (%)	11 (3.7)
CABG + MVR + tricuspid valve repair, n (%)	1 (0.3)
CABG + Bentall, n (%)	4 (1.3)
CABG + Bentall + mitral valve repair, n (%)	1 (0.3)
CABG + Bentall + tricuspid valve repair, n (%)	1 (0.3)
CABG + ventricular aneurysmectomy, n (%)	1 (0.3)
CABG + AVR + ascending aorta and aortic arch replacement, n (%)	1 (0.3)
CABG + AVR + ascending aorta replacement, n (%)	1 (0.3)
CABG + MVR + AVR + ascending aorta remodeling/replacement, n (%)	3 (1.0)
*Operative details*	
CABG off-pump, n (%)	7/214 (3.3)
Number of distal anastomoses	
1, n (%) 2, n (%) 3, n (%) 4, n (%) 5, n (%) 6, n (%)	67 (22.3) 125 (41.7) 74 (24.7) 28 (9.3) 5 (1.7) 1 (0.3)
Left internal mammary artery graft, n (%)	270 (90)
Right internal mammary artery graft, n (%)	10 (3.3)
Saphenous vein graft, n (%)	249 (83)
Number of surgical procedures	
Isolated CABG, n (%) 2 procedures, n (%) 3 procedures, n (%)	209 (69.7) 79 (26.3) 12 (4.0)
Cardiopulmonary bypass time, minutes, median [Q1-Q3]	106 [78–133]
Aortic cross clamp time, minutes, median [Q1-Q3]	65 [47–99]
*Post-operative details*	
Post-operative creatinine, mg/dl, median [Q1-Q3]	0.99 [0.82–1.23]
Post-operative hs-cTn I, ng/L, median [Q1-Q3] (patients)	
0 h 3–6 h 9–12 h 15–18 h 21–24 h 33–36 h 39–48 h	1353 [674–2469] (299) 2299 [1383–4242] (289) 2481 [1529–5409] (280) 2109 [1094–5156] (251) 1592 [814–3945] (257) 1057 [561–2940] (248) 1064 [458–3328] (162)
ECG criteria (new Q wave), n (%)	6 (2.0)
Echocardiographic criteria (new regional wall motion abnormalities), n (%)	19/299* (6.4)
Post-operative evidence of thrombotic occlusion of the graft and/or native coronary artery, n (%)	3 (1.0)
PMI, n (%)	24 (8.0)
Post-operative LVEF, %, median [Q1-Q3], (patients)	60 [50–64] (299)
Post-operative LVEF decrease ≥ 10%, n (%)	32/299* (10.7)
In-hospital composite morbidity after surgery	
Stroke, n (%)	3 (1.0)
Need for renal replacement therapy, n (%)	9 (3)
IABP/ECMO support, n (%)	6 (2.0)
Reintervention, n (%)	7 (2.3)
In hospital all-cause death, n (%)	5 (1.7)
Time from intervention to discharge/transfer, days, median [Q1–Q3]	9 [7–13]

AVR: aortic valve replacement; CABG: coronary artery bypass grafting; ECMO: extracorporeal membrane oxygenation; hs-cTn I: high-sensitivity cardiac troponin I; IABP: intra-aortic balloon pump; LVEF: left ventricular ejection fraction; MVR: mitral valve replacement; PCI: percutaneous coronary intervention; PMI: periprocedural myocardial infarction

*One patient died before post-operative echocardiography could be performed

### Post-operative details and outcomes

Hs-cTn I was serially measured for each patient, immediately at arrival to the Intensive Care Unit (defined as time 0) and then at regular intervals 3-to-6 h apart up to 39–48 h. Median hs-cTn I values and IR are showed in Table 2. The highest hs-cTn I values were measured at 9–12 h with a median of 2481 ng/L and IR of 1529–5409 ng/L.

Post-operative outcomes are showed in Table 2 as well. Five patients (1.7%) died; one death was due to cardiovascular causes (i.e., hemorrhagic stroke) while the remaining 4 were attributed to non-cardiovascular causes. A total of 24 patients (8.0%) developed a PMI, based on troponin elevation and at least one among electrocardiographic (6, 2.0%), echocardiographic (19, 6.4%) or angiographic (3, 1.0%) criteria. Three patients (1.0%) required urgent PCI after CABG, while 7 (2.3%) underwent reintervention. A total of 6 patients (2.0%) needed a mechanical circulatory support, in terms of IABP and/or ECMO. Renal replacement therapy was necessary in 9 cases (3%), while the remaining post-surgical morbidity was attributable to stroke (3 patients, 1.0%). The median post-operative LVEF was 60% (IR 50–64), and an LVEF decrease ≥ 10% was observed in 32 patients (10.7%). The median hospital length to discharge or transfer to another hospital consisted of 9 days (IR 7–13).

### Relationship between hs-cTn I and post-operative clinical outcomes

**Fig. 1:**
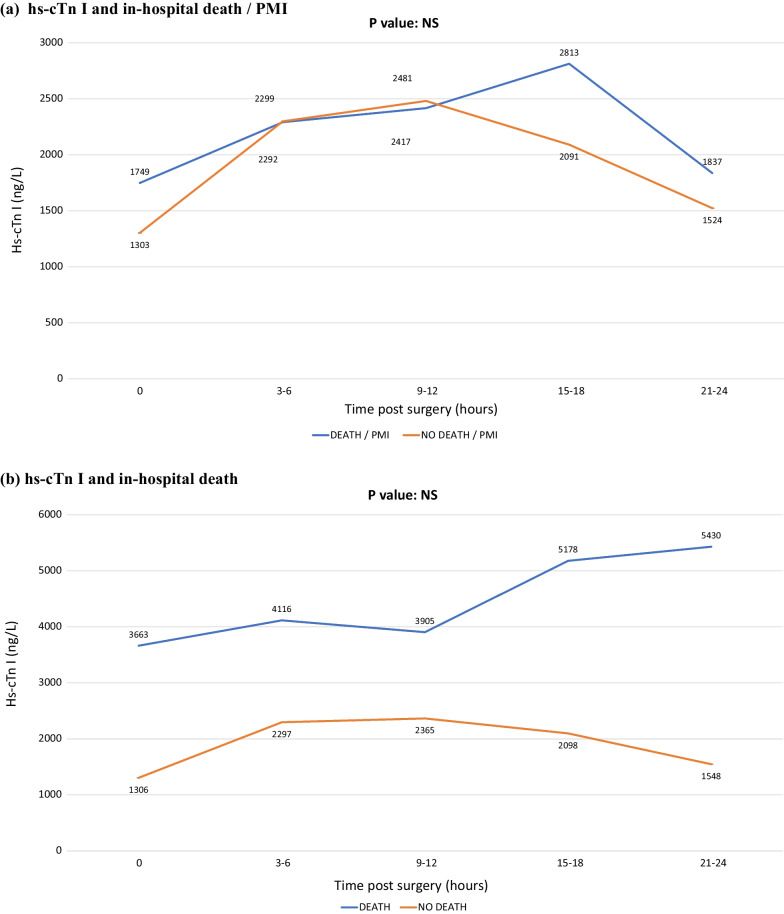

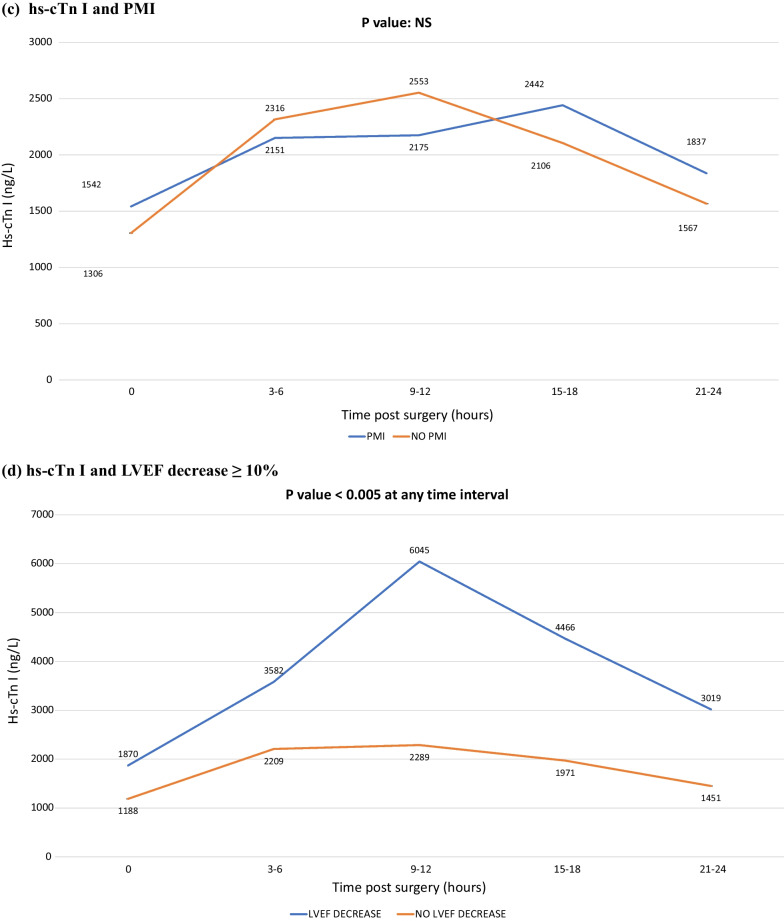
Relationship between post-operative hs-cTn I serial values and clinical outcomes. **A**: In-hospital death / PMI. **B**: In-hospital death. **C**: PMI. **D**: LVEF decrease ≥ 10%. hs-cTn I: high-sensitivity cardiac troponin I; LVEF: left ventricular ejection fraction; PMI: peri-operative myocardial infarction

Considering the numerosity of hs-cTn I values available at each time interval, we decided to analyze the ones collected up to 24 h after the end of the surgery, while discarding any subsequent value as those would be not representative enough of the whole category to allow for reliable considerations. The results of such analyses are shown in Additional file 1 Table S1 and in Fig. 1.

For any given time interval, we found no differences in hs-cTn I values with respect to the combined endpoint of in-hospital mortality or PMI (Fig. 1A), nor with in-hospital mortality (Fig. 1B), nor with PMI (Fig. 1C). In other words, the amount of troponin release was similar irrespectively of whether patients had died or not as well as irrespectively of whether they had developed a PMI or not. Conversely, hs-cTn I values were significantly higher in patients who showed a LVEF decrease ≥ 10% (32 patients, 10.7%) compared to patients who did not (Fig. 1D, *p* value < 0.005 at any time interval).

The univariate analysis (Table 3) including any variable that resulted significantly associated with post-operative LVEF decrease ≥ 10% at Wilcoxon rank-sum and Chi-squared tests (Additional file 1 Table S2) showed that post-operative LVEF decrease ≥ 10% was predicted by higher hs-cTn I values at 9–12 h post-operatively, along with previous cardiac surgery, higher number of surgical procedures, longer cardiopulmonary bypass time and aortic cross clamp time, evidence of new regional wall motion abnormalities at post-operative echocardiogram and PMI diagnosis. Moreover, we performed a ROC curve and identified, with the Youden's index method, a cutoff which is 5556 ng/L at 9–12 h post-operatively (a value 281 times the URL for male patients and 479 times the URL for female patients), representing the best balance between sensitivity (55%) and specificity (79%) in predicting LVEF decrease ≥ 10%.

**Table 3:** Univariate analysis for post-operative LVEF decrease ≥ 10%

	OR (95% CI)	*P* value
Post-operative hs-cTn I 0 h, for each 1000 ng/L	1.07 (0.99–1.16)	0.052
Post-operative hs-cTn I 3–6 h, for each 1000 ng/L	1.07 (0.98–1.16)	0.136
Post-operative hs-cTn I 9–12 h, for each 1000 ng/L	1.02 (1–1.05)	0.046
Post-operative hs-cTn I 15–18 h, for each 1000 ng/L	1.01 (0.99–1.02)	0.102
Post-operative hs-cTn I 21–24 h, for each 1000 ng/L	1.02 (0.99–1.04)	0.062
Previous cardiac surgery	17.73 (1.56–201.42)	0.02
*Number of surgical procedures*		
Isolated CABG 2 procedures 3 procedures	Reference 3.6 (1.6–7.9) 7.5 (2–28.4)	– 0.002 0.003
Cardiopulmonary bypass time, for each min increase	1.01 (1.01–1.02)	0.001
Aortic cross clamp time, for each min increase	1.02 (1.01–1.02)	< 0.001
Echocardiographic criteria (new regional wall motion abnormalities)	5.95 (2.15–16.48)	0.001
PMI	5.23 (2.03–13.47)	0.001

CABG: coronary artery bypass grafting; hs-cTn I: high-sensitivity cardiac troponin I; LVEF: left ventricular ejection fraction; PMI: periprocedural myocardial infarction

## Conclusions and discussion

Our study revealed several interesting findings about the prognostic value of hs-cTn I (Access, Beckman Coulter) after CABG surgery, alone or in association to other cardiac procedures. Firstly, we showed that post-operative hs-cTn I is so sensitive that in the vast majority of patients undergoing CABG surgery (i.e. 285/299 at 0 h, 254/257 at 24 h, 160/162 at 48 h) the hs-cTn I absolute value within 48 h after the intervention is superior to the limit required by the latest guidelines for the diagnosis of type 5 MI (10 times the 99^th^ percentile URL) [3]; of note, we observed the highest values at 9–12 h after surgery. Secondly, for any given time interval, we found in our population no significant differences in hs-cTn I absolute values with respect to in-hospital mortality or PMI. Thirdly, hs-cTn I values at 9–12 h post-operatively were significantly higher in patients who suffered a LVEF decrease ≥ 10% with respect to patients who did not (p value 0.046 at univariate analysis); actually, the majority of patients with such an echocardiographic finding had a hs-cTn I at 9–12 h higher than 5556 ng/L (as identified with the Youden's index method), a value 281 (for males) to 479 (for females) times higher than the URL.

When compared with previous myocardial laboratory markers like creatine kinase-MB (CK-MB), cTn have greater sensitivity and specificity in detecting myocardial necrosis, and have been found to be superior to CK-MB in predicting both in-hospital and long-term mortality in ACS and after CABG surgery [17, 18]. On the other hand, assays based on hs-cTn are more sensitive and accurate than “standard” cTn assays and therefore they are recommended by the most recent guidelines for the diagnosis of MI [5]. Despite the clear association between isolated elevations of “standard” I or T cTn with short-and long-term mortality after CABG surgery [19], sufficient data is currently lacking in the setting of post-CABG to accurately prove such an association between the main clinical adverse events and the absolute values of hs-cTn T or hs-cTn I. For this reason, the latest guidelines require so far additional ECG and/or imaging evidence of MI to identify those CABG patients at higher risk of mortality when a hs-cTn elevation > 10 times the URL is measured [3].

Given the aforementioned lack of evidence and the differences between the commercially available hs-cTn I assays, we focused our analysis on the values of hs-cTn I used in our Institution (Access hs-cTn I assay, produced by Beckman Coulter) in a population of 300 patients after CABG surgery, with serial measurements of hs-cTn I during the first 24 h, along with the analysis of complete echocardiographic and electrocardiographic post-operative data.

In line with previous authors [20], among post-operative CABG patients we did not find a significant association between hs-cTn I absolute values and mortality or PMI, although mean hs-cTn I values were more than double in patients who died in-hospital; it is also possible that the lack of statistical significance was due to the low number of main adverse clinical events. Furthermore, the absence in our study of the significant association between hs-cTn I absolute values and death or PMI showed by others authors with different hs-cTn assays [21] may be due to the fact that the superior sensitivity of the hs-cTn I assay has compromised its specificity and positive predictive value in the clinical setting of CABG patients.

Conversely, we found that hs-cTn I values were significantly higher in patients who showed a LVEF decrease ≥ 10% (32 patients, 10.7%) with respect to patients who did not (p value < 0.05 at any time interval). Moreover, at univariate analysis, among the different hs-cTn I values, only the one at 9–12 h post-operatively was associated to LVEF decrease ≥ 10%, along with other clinical factors (previous cardiac surgery, number of surgical procedures, cardiopulmonary bypass time, aortic cross clamp time, new echocardiographic regional wall motion abnormalities, and PMI diagnosis). Besides, we performed a ROC curve and identified a hs-cTn I cutoff value, equal to 5556 ng/L at 9–12 h post-operatively, representing the best balance between sensitivity (55%) and specificity (79%) in predicting a LVEF decrease ≥ 10%.

We evaluated the correlation between hs-cTn I values and a LVEF decrease > 10% after CABG surgery, considering the decrease in LVEF as a possible and reliable instrumental expression of myocardial damage while taking into account the well-known intra-observer and inter-observer variability of standard echocardiography [22]. Actually, one of the main findings of this study is that hs-cTn may be a useful, simple and objective method to identify, in the first day after CABG surgery, patients with decreased LVEF, and to guide emergent/urgent investigation and management of post-operative complications, potentially improving post-operative outcomes.

Another interesting finding of the study is that in the first 24 h after CABG surgery it could be possible to routinely perform only one basal and one second “9–12 h” hs-cTn I measurement, saving time and resources without losing any significant diagnostic or prognostic information.

Finally, to our knowledge, this is the first study specifically conducted on the hs-cTn I Access assay (produced by Beckman Coulter) in post-CABG patients and we confirm that, in this clinical setting, isolated hs-cTn I values may have a high sensitivity and low specificity for main cardiac adverse events as previously found with hs-cTn T [20].

The results of the present study have definite limitations. The whole data set of this retrospective, observational study comes from hospital records of a single center tertiary hospital. Moreover, we analyzed only hs-cTn I values in the first 24 h after CABG surgery, because we believed that the best time window for a meaningful improvement of the patients’ postoperative prognosis is the first post-operative day, as well as because after this time interval hs-cTn I values were no more regularly measured for all patients in our series, not allowing for a reliable complete analysis. Finally, our modest cohort size might have underpowered the analysis of some of the outcomes, which, as already underlined, could explain the lack of a significant association between hs-cTn I absolute values and mortality and/or PMI.

In summary, our study is a contemporary and detailed analysis of the prognostic performance of hs-cTn I assessed within 24 h after CABG surgery. We found that in our population hs-cTn I did not correlate with main cardiac adverse events, but higher hs-cTn I values at 9–12 h were predictors of post-operative LVEF decrease, and as such they may be useful to early identify patients at risk for LVEF decrease and to guide early investigation and management of possible post-operative complications. Given the high sensitivity and low specificity of hs-cTn I in the setting of post-CABG patients, our data support the current recommendation of always integrating hs-cTn I values with clinical, electrocardiographic and echocardiographic data for the diagnosis of type 5 MI.

## Supplementary Information

**Additional file 1.** Supplementary materials.

## Data Availability

The datasets used and/or analysed during the current study are available from the corresponding author on reasonable request.

## Abbreviations

ACS: Acute coronary syndrome
CABG: Coronary artery bypass graft
CI: Confidence interval
CK-MB: Creatine kinase-MB
ECG: Electrocardiogram
hs-cTn: High-sensitivity cardiac troponin
IABP: Intra-aortic balloon pump
IR: Interquartile range
LVEF: Left ventricular ejection fraction
MI: Myocardial infarction
OR: Odds ratio
PCI: Percutaneous coronary intervention
PMI: Periprocedural myocardial infarction
SD: Standard deviation
URL: Upper reference limit
