# Supplementary material for: Prognostic value of high-sensitivity cardiac troponin I early after coronary artery bypass graft surgery
Source: J Cardiothorac Surg. 2022 Nov 1;17:280. doi: 10.1186/s13019-022-02027-x (PMC9628166; doi:10.1186/s13019-022-02027-x)
Supplement: Supplementary file 1 — Additional file 1. Supplementary materials. [file 13019_2022_2027_MOESM1_ESM.docx]

**Additional file 1: Table S1. Post-operative hs-cTn I values and outcomes**

|  | **IN-HOSPITAL DEATH/PMI**  **(N=29)** | **NO IN-HOSPITAL DEATH/PMI**  **(N=271)** | **P value** |
| --- | --- | --- | --- |
| Post-operative hs-cTn I 0 hours, ng/L, median | 1749 (28) | 1303 (271) | 0.904 |
| Post-operative hs-cTn I 3-6 hours, ng/L, median | 2292 (28) | 2299 (261) | 0.967 |
| Post-operative hs-cTn I 9-12 hours, ng/L, median | 2417 (28) | 2481 (252) | 0.909 |
| Post-operative hs-cTn I 15-18 hours, ng/L, median | 2813 (24) | 2091 (227) | 0.168 |
| Post-operative hs-cTn I 21-24 hours, ng/L, median | 1837 (25) | 1524 (232) | 0.261 |
|  | **IN-HOSPITAL DEATH**  **(N=5)** | **NO IN-HOSPITAL DEATH**  **(N=295)** | **P value** |
| Post-operative hs-cTn I 0 hours, ng/L, median | 3663 (4) | 1306 (295) | 0.217 |
| Post-operative hs-cTn I 3-6 hours, ng/L, median | 4116 (4) | 2297 (285) | 0.389 |
| Post-operative hs-cTn I 9-12 hours, ng/L, median | 3905 (5) | 2365 (275) | 0.175 |
| Post-operative hs-cTn I 15-18 hours, ng/L, median | 5178 (4) | 2098 (247) | 0.101 |
| Post-operative hs-cTn I 21-24 hours, ng/L, median | 5430 (4) | 1548 (253) | 0.138 |
|  | **PMI**  **(N=24)** | **NO PMI**  **(N=276)** | **P value** |
| Post-operative hs-cTn I 0 hours, ng/L, median | 1542 (24) | 1306 (275) | 0.694 |
| Post-operative hs-cTn I 3-6 hours, ng/L, median | 2151 (24) | 2316 (265) | 0.682 |
| Post-operative hs-cTn I 9-12 hours, ng/L, median | 2175 (23) | 2553 (257) | 0.435 |
| Post-operative hs-cTn I 15-18 hours, ng/L, median | 2442 (20) | 2106 (231) | 0.460 |
| Post-operative hs-cTn I 21-24 hours, ng/L, median | 1837 (20) | 1567 (236) | 0.585 |
|  | **POST-OPERATIVE LVEF DECREASE**  **≥ 10%**  **(N=32)** | **NO POST-OPERATIVE LVEF DECREASE**  **≥ 10%**  **(N=267*)** | **P value** |
| Post-operative hs-cTn I 0 hours, ng/L, median | 1870 (32) | 1188 (267*) | 0.002 |
| Post-operative hs-cTn I 3-6 hours, ng/L, median | 3582 (29) | 2209 (260) | 0.003 |
| Post-operative hs-cTn I 9-12 hours, ng/L, median | 6045 (31) | 2289 (248) | 0.004 |
| Post-operative hs-cTn I 15-18 hours, ng/L, median | 4466 (29) | 1971 (222) | 0.001 |
| Post-operative hs-cTn I 21-24 hours, ng/L, median | 3019 (31) | 1451 (226) | 0.003 |

hs-cTn I: high-sensitivity cardiac troponin I; LVEF: left ventricular ejection fraction; PMI: periprocedural myocardial infarction.

* One patient died before post-operative echocardiography could be performed.

**Additional file 1: Table S2. Characteristics of the study population in patients with or without post-operative LVEF decrease ≥ 10%**

|  | **POST-OPERATIVE LVEF DECREASE**  **≥ 10%**  **(N=32)** | **NO POST-OPERATIVE LVEF DECREASE**  **≥ 10%**  **(N=267*)** | **P value** |  |
| --- | --- | --- | --- | --- |
| **Demographics and past medical history** | | | | |
| Age, years, median [Q1-Q3] | 71 [68-77] | 69 [63-76] | 0.184 |  |
| Males, n (%) | 24 (75.0) | 228 (85.4) | 0.129 |  |
| BMI, kg/m^2^, median [Q1-Q3] | 26.7 [24.1-30.8] | 26.8 [24.2-29.4] | 0.989 |  |
| Hypertension, n (%) | 21 (65.6) | 215 (80.5) | 0.065 |  |
| Hypercholesterolemia, n (%) | 21 (65.6) | 211 (79) | 0.114 |  |
| Diabetes, n (%)  on insulin, n (%) | 8 (25.0)  1 (3.1) | 87 (32.6)  13 (4.9) | 0.675 |  |
| Smoker  current, n (%)  previous, n (%) | 16 (50.0)  3 (9.4)  13 (40.6) | 166 (62.2)  42 (15.7)  124 (46.4) | 0.357 |  |
| Previous myocardial infarction, n (%) | 2 (6.3) | 56 (21.0) | 0.056 |  |
| Previous PCI/CABG, n (%) | 2 (6.3) | 45 (16.9) | 0.195 |  |
| Previous episode of congestive heart failure, n (%) | 3 (9.4) | 40 (15.0) | 0.593 |  |
| Previous stroke/TIA, n (%) | 2 (6.3) | 21 (7.9) | 1 |  |
| Peripheral arterial disease, n (%) | 8 (25.0) | 49 (18.4) | 0.348 |  |
| Symptomatic chronic lung disease, n (%) | 1 (3.1) | 17 (6.4) | 0.704 |  |
| Dialysis, n (%) | 1 (3.1) | 3 (1.1) | 0.366 |  |
| **Pre-operative clinical characteristics** | | | | |
| Hospitalization for ACS, n (%) | 10 (31.3) | 90 (33.7) | 0.845 |  |
| Left main stenosis >50%, n (%) | 5 (15.6) | 68 (25.5) | 0.279 |  |
| Three-vessel disease, n (%) | 12 (37.5) | 135 (50.6) | 0.192 |  |
| Creatinine, mg/dl, median [Q1-Q3] | 0.94 [0.81-1.02] | 0.96 [0.80-1.14] | 0.252 |  |
| **Other pre-operative risk assessment parameters** | | | | |
| Previous cardiac surgery, n (%) | 2 (6.3) | 1 (0.4) | 0.031 |  |
| Active IE, n (%) | 1 (3.1) | 2 (0.7) | 0.289 |  |
| Non-elective operation, n (%) | 11 (34.4) | 119 (44.6) | 0.506 |  |
| EuroScore II estimated risk of in-hospital mortality, mean ± SD | 3.2 ± 3.9 | 2.9 ± 4.9 | 0.735 |  |
| **Operative details** | | | | |
| CABG off-pump, n (%) | 0 (0) | 7 (2.6) | 1 |  |
| Number of distal anastomoses ≤ 3, n (%) | 29 (90.6) | 236 (88.4) | 0.935 |  |
| Number of surgical procedures  Isolated CABG  2 procedures  3 procedures | 13 (40.6)  15 (46.9)  4 (12.5) | 196 (73.4)  63 (23.6)  8 (3.0) | < 0.001 |  |
| Cardiopulmonary bypass time, minutes, median [Q1-Q3] | 134 [103-171] | 103 [76-130] | < 0.001 |  |
| Aortic cross clamp time, minutes, median [Q1-Q3] | 104 [62-138] | 64 [43-90] | < 0.001 |  |
| **Post-operative details** | | | | |
| Post-operative creatinine, mg/dl, median [Q1-Q3] | 0.94 [0.81-1.21] | 0.99 [0.82-1.23] | 0.516 |  |
| Post-operative hs-cTn I, ng/L, median [Q1-Q3], (patients)  0 hours  3-6 hours  9-12 hours  15-18 hours  21-24 hours | 1870 [1330-3941]  3582 [2233-6133]  6045 [1810-9194]  4466 [2194-8913]  3019 [1101-9263] | 1188 [654-2383]  2209 [1331-4066]  2289 [1432-4757]  1971 [1073-4586]  1451 [789-3342] | 0.002  0.003  0.004  0.001  0.003 |  |
| ECG criteria (new Q wave), n (%) | 2 (6.3) | 4 (1.5) | 0.127 |  |
| Echocardiographic criteria (new regional wall motion abnormalities), n (%) | 7 (21.9) | 12 (4.5) | 0.002 |  |
| Post-operative evidence of thrombotic occlusion of the graft and/or native coronary artery, n (%) | 0 (0) | 3 (1.1) | 1.000 |  |
| PMI, n (%) | 8 (25.0) | 16 (6.0) | 0.002 |  |
| In-hospital composite morbidity after surgery, n (%)  stroke, n (%)  need for renal replacement therapy, n (%)  IABP/ECMO support, n (%)  reintervention, n (%) | 1 (3.1)  1 (3.1)  0  0 | 1 (0.4)  8 (3.0)  6 (2.2)  7 (2.6) | 0.203  1  0.85  1 |  |

* One patient died before post-operative echocardiography could be performed.

**FIG.1 (A TO D) HEADING:**

**Relationship between post-operative hs-cTn I serial values and clinical outcomes**

**FIG.1 (A TO D) SUBHEADINGS:**

**A: In-hospital death / PMI**

**B: In-hospital death**

**C: PMI**

**D: LVEF DECREASE ≥ 10%**

**FIG.1 (A TO D) LEGEND:**

hs-cTn I: high-sensitivity cardiac troponin I; LVEF: left ventricular ejection fraction, PMI: peri-operative myocardial infarction.
